# Differences between the Filling Velocities of the Left and Right Heart Ventricle in Racing Pigeons (*Columba livia* F. Domestica) and the Influence of Anesthesia with Isoflurane

**DOI:** 10.3390/vetsci6040079

**Published:** 2019-10-09

**Authors:** Marko Legler, Lajos Koy, Norbert Kummerfeld, Michael Fehr

**Affiliations:** Clinic for Small Mammals, Reptiles and Birds, University of Veterinary Medicine Hannover, Foundation, Bünteweg 9, D-30559 Hannover, Germany; lajos.schmitt@gmx.de (L.K.); nokumf@gmx.de (N.K.); michael.fehr@tiho-hannover.de (M.F.)

**Keywords:** sonography, heart valve, blood flow velocity, diastole, birds

## Abstract

The ventricular filling velocities during diastole and the influence of isoflurane anesthesia on these blood flow velocities of the racing pigeon (*n* = 43) are evaluated by pulsed-wave (PW) Doppler sonography. Sonographic examination demonstrates an early passive ventricular (E wave) and late active (A wave) ventricular filling. The results indicate differences between the two heart ventricles. Especially, the E wave velocity of the right heart is significantly lower than in the left heart, which is explained by the crescent-shaped cavity of the right ventricle around the left ventricle. The faster active filling velocities are significantly influenced by heart rate in conscious birds. Anesthesia with isoflurane leads to a significant decrease of the diastolic blood flow velocities, and the A wave velocities of both ventricles are especially influenced. Anesthesia with isoflurane induces a high incidence of insufficiencies of the left atrioventricular valve in the preejection period. These observations indicate that a contraction of the left ventricle myocardium is important for a complete valvular closure and for normal functioning of this heart valve. The effective closure of the right atrioventricular muscle valve in anesthetized pigeons supports the observation of the fast innervation of this muscle valve via a direct connection to the right atrium.

## 1. Introduction

The avian heart is four-chambered, has two completely separated atria and ventricles, and is functionally comparable to the mammalian heart [1,2]. The thick-walled left ventricle is cone-shaped and extends to the apex of the heart. In contrast, the thin-walled right ventricle does not reach the apex and lies crescent-shaped around the left ventricle. Unique to avian species is the anatomy of the atrioventricular (AV) valves. The left AV valve is formed as a membranous tricuspid AV valve, similar to the anatomically structure of mammalian species [1,2]. In contrast, the right AV valve is an oblique muscular flap of the right-ventricular free wall and consists of atrial and ventricular myocardial parts [1,3]. The right AV is closely linked to an atrioventricular ring bundle of pacemaker and conducting myocytes (formerly known as AV-Purkinje ring), an important part of the conducting system of the avian heart [1,3,4,5,6]. The function of the left AV valve is the passive closure of the AV orifice depending on the pressure gradient [1,2]. In contrast, the muscular structure of the right AV valve allows for the active closure of the right AV orifice and the participation of this valve in the pumping function of the right ventricle [3,6,7]. The right atrium is compared to the left atrium larger [2,8,9]. The diastolic function in avian species is in detail inconsistent in the literature. The existence of an early diastolic ventricular blood inflow (E wave) and an active ventricular filling (A wave) has been described in different avian species, such as mammals [8,9,10]. However, the results in the literature indicate that the ratio of E to A waves varies between different avian species, and in higher heart rates E and A waves are fused and become a single EA wave [9,10]. The majority of avian echocardiography studies have been conducted under isoflurane anesthesia [11,12,13,14]. The anesthetic isoflurane allows for easily controllable and safe anesthesia in birds and is often used to enable an adequate clinical examination [15,16,17,18]. In small animal medicine, the influence of anesthesia on cardiac parameters has been well described [19,20,21,22,23,24,25,26]. Little is known about the influence of isoflurane on Doppler-derived blood flow velocities, flow pattern, and myocardial or heart valve functions in avian species [27]. The aim of the present study is to examine the ventricular filling during the diastole and to evaluate the possible influence of isoflurane anesthesia on these blood flow velocities and the AV function of the heart in racing pigeons measured by pulsed-wave (PW) Doppler sonography. 

## 2. Materials and Methods

The study was conducted in accordance with the German animal welfare regulations and with the permission of the relevant German authorities (reference number: 33.12-42502-04-15/1864). 

### 2.1. Experimental Animals

Racing pigeons (*Columba livia* f. dom.; *n* = 43) of both sexes (male: *n* = 16; female: *n* = 27) were used for the investigations. The pigeons were 2.30 ± SD 1.69 (range: 0.5 to 8) years old and had a weight of 468.16 ± SD 51.64 (range: 352–577g) g body mass and were trained for racing. The sternal length of the pigeons measured from the visible sternocoracoid joint to the end of the sternum in laterolateral radiographic images was 73.4 millimeter (mm) ± SD 3.1 mm (range: 63.2–81.0 mm). The pigeons were housed in indoor aviaries, being offered a commercial pigeon seed mix and fresh drinking water ad libitum and were routinely vaccinated by the owner for pigeon avulavirus 1 (paramyxovirus 1) and salmonellosis. All pigeons showed normal feeding and drinking behavior. Prior to the ultrasound examinations, the pigeons were acclimatized for two weeks in the new aviaries. During this time, bacteriological and parasitological testing was carried out of a composite fecal sample of the pigeons for salmonella and endoparasites of the intestine, which was negative. A microscopic examination of fresh crop samples revealed in some pigeons a low grade infestation with *Trichomonas gallinae*, and all pigeons were treated with 10 mg Carnidazole (Spatrix^®^, Elanco Deutschland GmbH). The pigeons were declared healthy by clinical, hematological, and radiological examinations. The maximum width of the cardiac silhouette of the pigeons measured in the ventrodorsal radiograph was 58.8% ± 3.3 (50.3–65.1%) of the maximum width of the thorax. These results are comparable to results of healthy birds in the literature [9]. The hematocrit (44.9% ± 1.8; 42.0–49.0%) and the buffy coat (˂1%) of the pigeons were in the reference values of healthy and normal hydrated pigeons [28]. 

Egg-laying pigeons were excluded from sonographic examinations, to prevent the influence of higher abdominal pressure.

### 2.2. Doppler-Sonographic Examination

Echocardiographic images were acquired by using a 10 MHz linear-array transducer (B Mode 4.5–11.5 MHz) with a digital ultrasound system (Vivid 7 Dimension BT08, GE Medical Systems) in combination with an electrocardiogram according to Einthoven [29,30,31]. The pigeons were fixed in a semi-upright position for the sonographic examination of the heart from the left and right parasternal approach. Depending on the individual bird, the left and right fenestra or the space behind the last rib through the liver to the heart were chosen as acoustic windows. The 2-D echocardiographic images were named and oriented in accordance with Riedel, 1995 [32]; Schulz, 1995 [33]; and Pees et al., 2006 [9]. The right parasternal longitudinal horizontal view was chosen to measure the right and left diastolic ventricular blood inflow. The blood flow velocities were estimated at the level of the AV valves by pulsed-wave (PW) Doppler sonography. Sonographic images were used in the evaluations that were generated with a setting of more than 80 frames per second. The sample volume was 1.5 mm. A wall filter setting of 3.4 cm/s was chosen. The angle correction cursor was used for every measurement, aligning it as parallel as possible to the direction of blood flow, and parallel to the interventricular septum.

Measurements of the diastolic blood flow were taken over six sequential individual heart beats in each PW Doppler image, and the mean was used for further evaluations. One PW Doppler image per location was analyzed of each pigeon. All measurements were performed with calipers on the frozen screen images using the on-board ultrasound system computer. The diastolic peak flow velocities in meter per second (m/s) were determined at the time of maximum blood flow velocity in E and A wave or EA wave (fused E and A wave).

Additionally, the diastolic blood flow was examined in the AV regions by color Doppler flow imaging to visualize valvular insufficiencies. The color Doppler flow images were obtained with setting of more than 80 frames per second. 

### 2.3. Anesthesia

In each racing pigeon, the echocardiographic examination was performed once in conscious birds and once under general anesthesia with isoflurane (Isofluran CP^®^, CP Pharma GmbH, Burgdorf, Germany) at a minimum interval of two days. The pigeons were prepared for anesthesia by withdrawing food for 4 hours and water for a half hour before anesthetic procedure. Anesthesia was induced with 4% isoflurane in 1 L/min oxygen and maintained with 1 to 2% isoflurane in 1 L/min oxygen with an anesthetic mask and spontaneous breath. The depth of anesthesia was calculated by toe pinch and wing twitch reflexes. The echocardiographic examination was performed during the stage of surgical anesthesia.

### 2.4. Statistical Analysis

Statistical tests were performed using SPSS^®^ Statistics 24. Mean, median, standard deviation (SD), and range (Xmin to Xmax) were calculated for the different parameters of the flow pattern of the left and right diastolic filling. The Kolmogorov–Smirnov test was used to test for normal distribution of measured values. According to these results, the student’s t test for matched pairs or the Wilcoxon signed-rank test, as well as the student’s unpaired t test or the Mann–Whitney U test, were chosen for the evaluations. The Spearman’s rank correlation coefficient was used to visualize the influence of the heart rate on parameters of aortic and pulmonary blood flow pattern of conscious and anaesthetized pigeons. A significance level of *p* ≤ 0.05 was chosen. To evaluate the incidence of valvular insufficiencies between conscious and anaesthetized pigeons, a chi–square test was performed.

## 3. Results

### 3.1. Diastolic Ventricular Filling Blood Flow Velocities in Conscious Racing Pigeons

**Figure 1:**
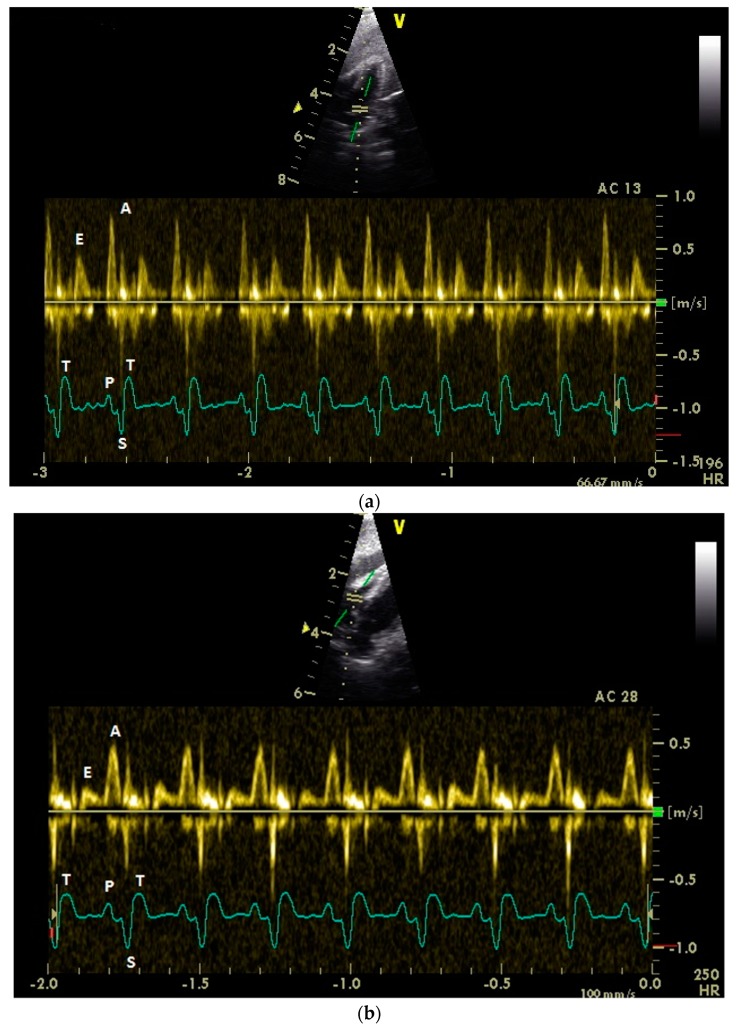
Pulsed-wave Doppler echocardiographic images of a conscious healthy racing pigeon of the left (**a**) and the right (**b**) atrioventricular blood flow using right parasternal approach. Diastolic blood flow: E: early passive ventricular filling; A: active ventricular filling. Electrocardiogram: P: P wave; S: S wave; and T: T wave.

Adequate measurements of the diastolic blood flow of the left and right heart (Figure 1) were possible in all examined 43 racing pigeons. 

A fused E and A wave (EA wave) were observed in two birds in the conscious pigeon group and in five birds in the anaesthetized pigeon group in a heart rate range of 270 to 360 bpm in conscious and 189 to 270 bpm in anaesthetized pigeons.

An overview of the detected diastolic velocities is shown in Table 1.

A significant difference between the velocities of the early diastolic ventricular filling (E wave) and the active ventricular filling (A waves) in the left and the right heart was observed. The A wave velocities were significant higher than the E wave velocities in both ventricles (*p* ≤ 0.001; Mann–Whitney U test). 

There was a significant difference between the E wave blood flow velocities of the left (0.37 ± 0.06) and right heart (0.22 ± 0.06; *p* ≤ 0.001; Mann–Whitney U test). The blood flow velocities of the A waves as well as EA waves were similar in the left and right heart (*p* > 0.25; Mann–Whitney U test; student’s *t* test).

There was a significant difference between the E to A ratio of the left and right heart (*p* ≤ 0.001; Mann–Whitney U test).

### 3.2. Influence of Isoflurane Anesthesia on Diastolic Blood Flow Velocities

Anesthesia with isoflurane leads to significantly reduced diastolic blood flow velocities (Table 1; *p* ≤ 0.05, Wilcoxon signed-rank test). 

In anesthetized pigeons, there were also significant differences between the lower E and higher A wave velocities in the left and in the right heart (*p* ≤ 0,001; Mann–Whitney U test). 

Under anesthesia, the E to A ratio was significantly higher for the left heart chamber (*p* = 0.017; Mann–Whitney U test).

### 3.3. Influence of Heart Rate (HR), Body Indices, Age, and Sex on the Diastolic Blood Flow Velocities 

**Figure 2:**
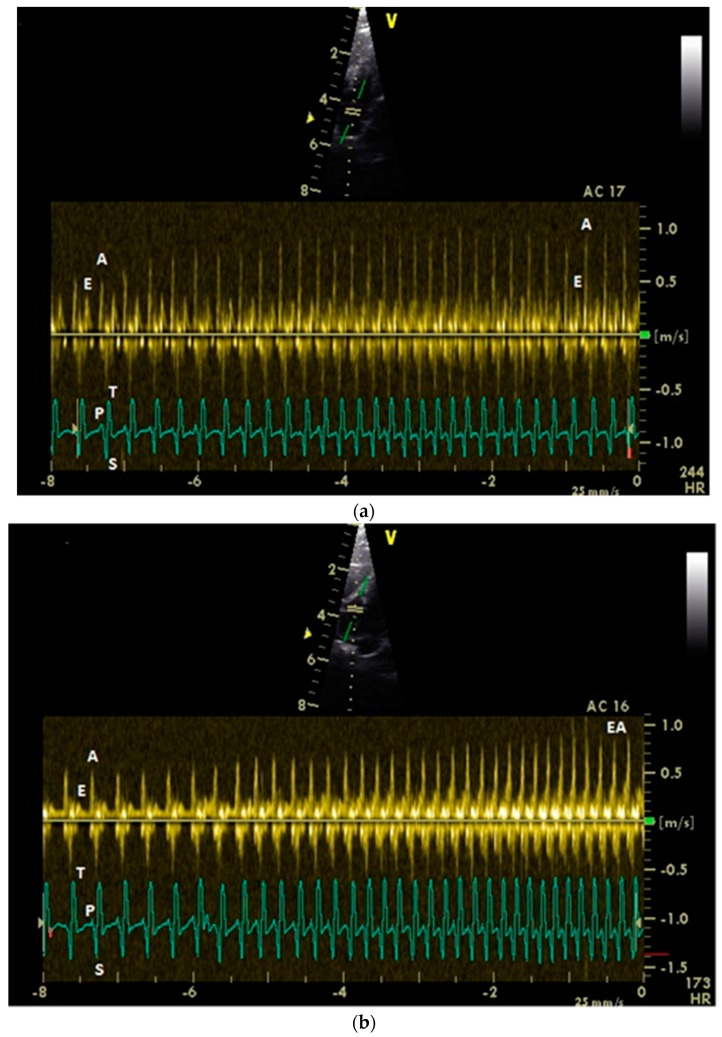
Pulsed-wave Doppler echocardiographic images of a conscious healthy racing pigeon of the left (**a**) and the right (**b**) atrioventricular blood flow depending on heart rate (left a: 166–244; right b: 173–240) using right parasternal approach. Diastolic blood flow: E: early passive ventricular filling; A: active ventricular filling; and EA: fused E and A wave. Electrocardiogram: P: P wave; S: S wave; and T: T wave.

The influence of the heart rate on the peak diastolic flow velocity is significant for the E wave in the right heart (*p* = 0.007, Spearman’s correlation coefficient *r* = 0.417, low correlation) and for the left and right A wave (*p* ≤ 0.001; Spearman’s correlation coefficient *r* = 0.527–0.535, middle correlation; Figure 2). There was no significant influence of the heart rate on left E wave diastolic filling (*p* = 0.97; Spearman’s correlation: *r* = −0.006).

The influence of the heart rate on the E to A wave ratio was significant in the left heart (*p* ≤ 0.001, Spearman’s correlation coefficient: *r* = −0.542, middle correlation). In contrast to these findings, there was no influence of the heart rate on the E to A wave ratio in the right heart (*p* = 0.42; Spearman’s correlation coefficient: *r* = −0.13).

There was a significant negative influence of the age on the early ventricular filling of the left ventricle (*p* = 0.046; Spearman’s correlation coefficient: *r* = −0.313 low correlation). A significant influence of the sex was not detected in our examinations (*p* > 0.17).

### 3.4. Examination of the Diastolic Blood Flow with Color Doppler Flow Imaging

**Figure 3:**
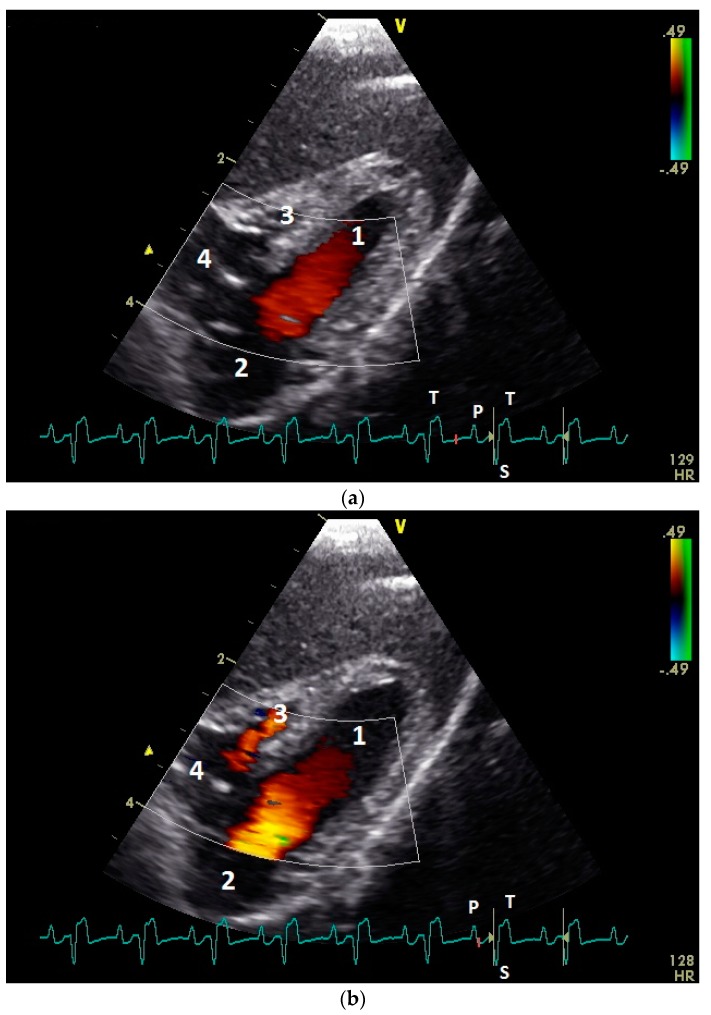
Color Doppler echocardiographic image of the early passive ventricular filling blood flow (**a**) and the late active ventricular blood inflow (**b**) of a conscious healthy racing pigeon using the right parasternal approach. 1: left ventricle; 2: left atrium; 3: right ventricle; and 4: right atrium. The red color visualizes the atrioventricular blood flow. Electrocardiogram: P: P wave; S: S wave; and T: T wave.

**Figure 4:**
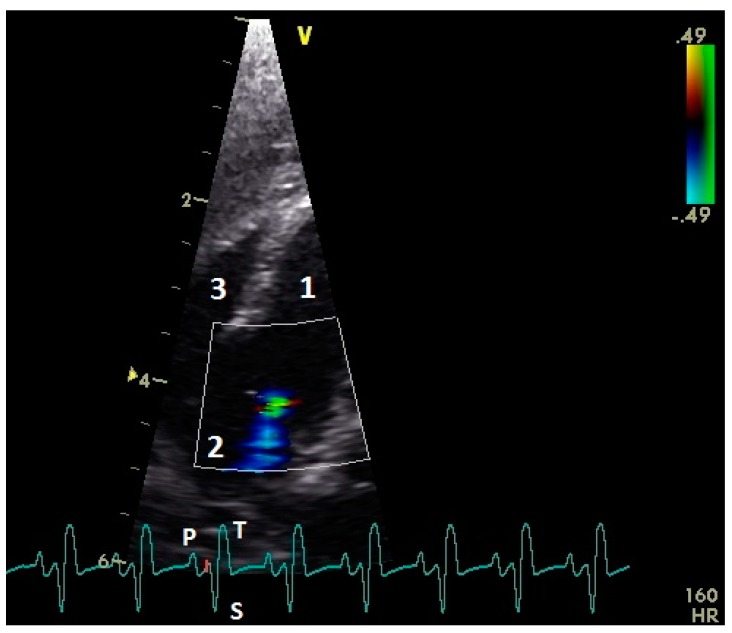
Color flow image of the left atrioventricular valve region of an anesthetized racing pigeon with a valvular insufficiency (blue color) in the preejection period after the active ventricular filling and before the systolic ventricular contraction (red mark on electrocardiogram). 1: left ventricle; 2: left atrium, and 3: right ventricle. Electrocardiogram: P: P wave; S: S wave; and T: T wave.

**Figure 5:**
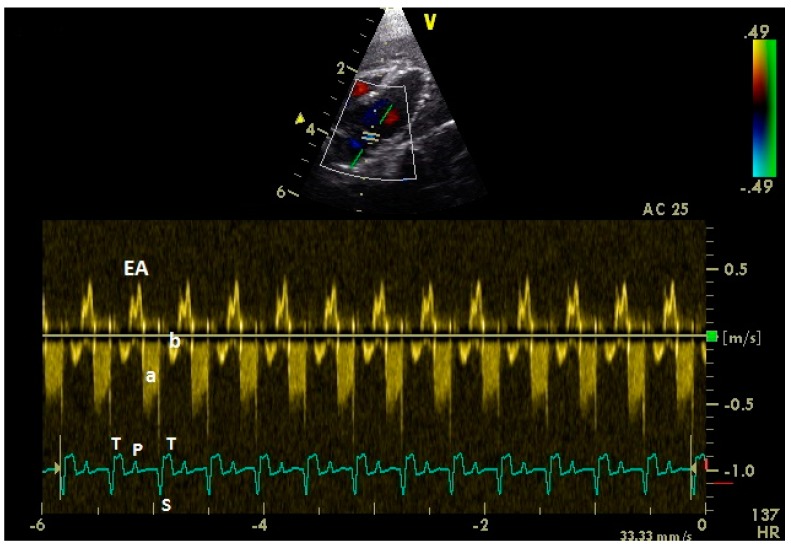
Pulsed-wave Doppler echocardiographic image of the left atrioventricular valve region of an anaesthetized healthy racing pigeon. Diastolic blood flow: EA: fused E and A wave. a: left atrioventricular insufficiency; b: part of systolic flow in left ventricle. Electrocardiogram: P: P wave; S: S wave; and T: T wave.

In color flow images of the atrioventricular valve regions, the early and the active ventricular filling synchronous to the ventricular relaxation and atrial contraction could be observed (Figure 3). The left AV valve leaflets opened in the early and after a short closing a second time in the active diastolic filling. Higher heart rates (EA waves, early and active ventricular filling combined) lead to only one opening of the left AV valve leaflets. The right AV muscle valve closed only immediately after the active diastolic filling. In the preejection period, between the end of the active diastolic filling and the contraction of the ventricle myocardium, we found in two awake pigeons (4.7%) a left AV valve insufficiency. In the group of the anaesthetized pigeons, 40 birds (93.0%) showed in this preejection period a left AV insufficiency (comparison conscious and anaesthetized pigeons: *p* ˂ 0.001, chi–square test; Figure 4 and Figure 5). One pigeon developed under anesthesia a holosystolic left AV insufficiency. Right AV insufficiencies were found only in the preejection period of 3 (7.0%) anesthetized pigeons (comparison conscious and anesthetized pigeons: *p* = 0.08, chi–square test). Thus, there is a significant difference between left and right AV closure in anesthesia (*p* ˂ 0.001, chi–square test). 

## 4. Discussion

The ventricular filling in avian species follows the same principles like in mammals with an early or passive diastolic filling (E wave) and an active filling during the atrial contraction (A wave; [9,34]). In our study, the E wave velocities in the examined pigeons are pretty constant and only slightly influenced by heart rate or anesthesia. An explanation of these constant values in our cases could be a low stress response through handling of the trained pigeons. In other animals, for example in dogs, different studies show different results for the influence of anesthesia on E Wave velocities [35,36,37,38]. However, contrasting to small animal cardiology, the results of our study demonstrate significant differences between the passive filling velocities of the left and right ventricle in birds, with significant lower E Wave velocities in the right heart. Possible explanations for these differences are the anatomical characteristics of birds. The right ventricle lies crescent-shaped around the left ventricle, and in the relaxation of the ventricles the right ventricle is more stretched [1,2]. Thus, the opening of the right ventricle is limited and the passive ventricular filling is reduced in contrast to the left ventricle. In contrast, the E wave velocities of tricuspidal and mitral blood flow in dogs are not significant different [35,36]. These findings also indicate that the atrial contraction for the filling of the right ventricle is very important. For this special ventricular filling, a great right atrium as blood reservoir for the active filling seems to be necessary [2]. In our study in pigeons, the A wave velocities are significant higher than E wave filling velocities. These findings are completely different from investigations in mammals. In small animal medicine, the higher A wave velocities are a sign of an increased preload in heart failure and/or ventricular relaxation abnormality [34,35,36,37,38,39,40]. In the literature for birds, there is different information about E and A wave velocities and the ratio of these two velocities [9,12,13,41]. Masoudifard et al. (2016) [42] found slightly lower ventricular inflow velocities in racing rigeons, left 0.49 and right 0.33 m/s, without differentiation between E and A wave velocity and angle correction. The significant influence of the heart rate on the A wave velocities of the left ventricular inflow led to a significant negative correlation of the heart rate and the E to A ratio. This influence of the heart rate is also seen in the variable A wave velocities of blood flow as well as in the maximal and minimal values of the ventricular filling velocities. These complex relationships could be a possible explanation for different data of the ratio of E to A wave in the literature. In most of the published studies, the velocities are evaluated in anesthesia. The different E to A ratios in various bird species could be a result of a different influence of anesthesia in these birds. In our study, anesthesia with isoflurane had a significant influence on the diastolic velocities of pigeons and led mainly to reduced diastolic blood flow velocities, especially of the A wave velocities, and to a changed E to A wave ratio in the left heart. This influence of anesthesia has also been described in mammals [26,38] and in other birds [12,27]. Peak flow velocities are sensitive indexes of myocardial contractility [38]. Different studies show that isoflurane depresses myocardial contractility in mammals [43]. Thus, anesthesia with isoflurane results in arterial blood pressure, cardiac output, and stroke volume decrease [18,44,45,46,47,48]. In higher heart rates, in our study from about 300 bpm in conscious pigeons, the E and A waves were fused to an EA wave. The relationship of heart rate and A wave velocities in our investigations indicate that the EA waves are mainly influenced by the velocities of the A wave. The heart rates in the conscious pigeons in our study should be interpreted as values at rest [49]. The birds were trained and accustomed to handling. The high SD reported for the heart rates under anesthesia in our study was attributed to pigeons with very low and with high baseline heart rates. Comparable data were observed in dogs under anesthesia with unknown cause [38]. It is remarkable that under anesthesia, EA waves are recognized in significantly lower heart rates. These findings could be a result of a reduced stroke volume and a higher end-systolic ventricular volume in combination with a prolonged relaxation time in anesthetized, compared to conscious, pigeons.

The examinations of the diastolic blood flow velocities with PW Doppler sonography and color Doppler imaging in anesthesia and in conscious pigeons allows one to attain insight into the AV valves function in birds. The high incidences of left AV insufficiencies in the preejection period indicate that the contraction of the left ventricular myocardium is important for complete left AV valve closure. A prolonged conduction of the contractile impulse in the atrioventricular conduction system in anesthesia led to a delayed ventricular myocardial contraction, and reflux of blood to the left atrium in this time between the diastole and systole could have occurred. In contrast to the observations on the left AV valve, a reflux of blood at the right valve could only be observed in a few of our cases under anesthesia; in most pigeons, the muscular right valve also worked very reliably under anesthesia. These observations show that even under anesthesia, the closure of the right AV valve occurs without time delay immediately after the active ventricular filling. Furthermore, Prosheva et al. (2015) describe in the chicken heart fast muscular valve closure much earlier than the ejection time of the right ventricle and a simultaneous electrical activation of the muscular valve and the main bulk of the right ventricular free wall [7,50]. A possible explanation of this fast valve closure could be the anatomy of the right muscular AV valve. The atrial muscular layer of the right AV valve could allow for direct electrical activation of parts or the whole muscular valve directly from the atria without delay, with rapid closure immediately upon atrial contraction. Our investigations in anesthesia suggest such an explanation, because electrical activation of the ventricular myocardium via the AV node region needs much more time in our cases. It is possible that the atrioventricular ring bundle of pacemaker and conducting cells in the muscular atrial layer of the right AV valve provide this rapid and uniform electrical excitation of this muscular valve [3,5,6,50]. 

In the clinical examination of birds in anesthesia, these insufficiencies in the preejection period can be misinterpreted as pathological valvular insufficiencies. Only one bird showed under anesthesia a holosystolic insufficiency of the left AV valve. However, more research is necessary to understand fast changing heart velocities and to establish reverence values for conscious individuals of different avian species.

## Figures and Tables

**Table 1:** Diastolic ventricular filling velocities (m/s) of conscious and anaesthetized healthy racing pigeon (mean ± standard deviation; median; and Xmin–Xmax).

Measuring Point	Peak E Velocity	Peak A Velocity	E : A Ratio	Peak EA Velocity	Angle Correction
Left AV valve	0.37 ± 0.59	0.58 ± 0.20	0.72 ± 0.28	0.84 ± 0.01	28.4 ± 7.8
	(0.37; 0.25–0.53)	(0.57; 0.24–0.96)	(0.64; 0.31–1.63)	(0.84; 0.83–0.85)	(29.0; 12.0–45.0)
Heart rate	220.5 ± 41.3			(270.0–360.0)	
	(225.0; 146.0–300.0)				
Left AV valve in anesthesia	0.34 ± 0.07 (0.33; 0.26–0.52)	0.42 ± 0.12 (0.41; 0.22–0.67)	0.87 ± 0.32 (0.78; 0.44–1.79)	0.55 ± 0.17 (0.55; 0.43–0.67)	27.4 ± 7.9 (28.0; 0.0–45.0)
Heart rate	148.4 ± 29.5			(210.0–257.0)	
	(146.0; 99.0–244.0)				
Right AV valve	0.22 ± 0.06	0.52 ± 0.10	0.43 ± 0.11	0.67 ± 0.01	22.2 ± 7.82
	(0.2; 0.12–0.42)	(0.51; 0.31–0.70)	(0.4; 0.25–0.71)	(0.67; 0.66–0.68)	(24.0; 8.0–40.0)
Heart rate	219.1 ± 34.7			(300–360)	
	(215.5; 148–300)				
Right AV valve in anesthesia	0.18 ± 0.06 (0.18; 0.03–0.38)	0.47 ± 0.11 (0.45; 0.24–0.80)	0.42 ± 0.24 (0.37; 0.09–1.58)	0.37 ± 0.05 (0.43; 0.36–0.50)	21.8 ± 7.6 (22.0; 0.0–35.0
Heart rate	148.7 ± 20.3			235.8 ± 31.6	
	(147.5; 100.0–197.0)			(240.0; 189.0–270.0)	
